# Clinical and molecular characterization of *HER2* amplified-pancreatic cancer

**DOI:** 10.1186/gm482

**Published:** 2013-08-31

**Authors:** Angela Chou, Nicola Waddell, Mark J Cowley, Anthony J Gill, David K Chang, Ann-Marie Patch, Katia Nones, Jianmin Wu, Mark Pinese, Amber L Johns, David K Miller, Karin S Kassahn, Adnan M Nagrial, Harpreet Wasan, David Goldstein, Christopher W Toon, Venessa Chin, Lorraine Chantrill, Jeremy Humphris, R Scott Mead, Ilse Rooman, Jaswinder S Samra, Marina Pajic, Elizabeth A Musgrove, John V Pearson, Adrienne L Morey, Sean M Grimmond, Andrew V Biankin

**Affiliations:** 1 Kinghorn Cancer Centre and Garvan Institute of Medical Research, Darlinghurst, Sydney, Australia; 2 Anatomical Pathology, Sydpath, St Vincent’s Hospital, Sydney, Australia; 3 St Vincent’s Clinical School, University of New South Wales, Sydney, Australia; 4 Department of Anatomical Pathology, Royal North Shore Hospital, St Lenoards, Sydney, Australia; 5 Sydney Medical School, University of Sydney, Sydney, Australia; 6 Queensland Centre for Medical Genomics, Institute for Molecular Bioscience, University of Queensland, Brisbane, Australia; 7 Department of Cancer Medicine, Hammersmith Hospital, Imperial College Healthcare NHS Trust, London, UK; 8 Prince of Wales Clinical School, University of New South Wales and Prince of Wales Hospital, Sydney, Australia; 9 Histopath Pathology, 97 Waterloo Road, North Ryde, NSW 2113, Australia; 10 Macarthur Cancer Therapy Centre, Sydney South West District Health Service, Sydney, NSW, Australia; 11 Upper Gastrointestinal Surgery Unit, Royal North Shore Hospital, Sydney, Australia; 12 Wolfson Wohl Cancer Research Centre, Institute of Cancer Sciences, University of Glasgow, Glasgow, UK; 13 West of Scotland Pancreatic Unit, Glasgow Royal Infirmary, Glasgow, UK

## Abstract

**Background:**

Pancreatic cancer is one of the most lethal and molecularly diverse malignancies. Repurposing of therapeutics that target specific molecular mechanisms in different disease types offers potential for rapid improvements in outcome. Although *HER2* amplification occurs in pancreatic cancer, it is inadequately characterized to exploit the potential of anti-*HER2* therapies.

**Methods:**

*HER2* amplification was detected and further analyzed using multiple genomic sequencing approaches. Standardized reference laboratory assays defined *HER2* amplification in a large cohort of patients (n = 469) with pancreatic ductal adenocarcinoma (PDAC).

**Results:**

An amplified inversion event (1 MB) was identified at the *HER2* locus in a patient with PDAC. Using standardized laboratory assays, we established diagnostic criteria for *HER2* amplification in PDAC, and observed a prevalence of 2%. Clinically, *HER2*- amplified PDAC was characterized by a lack of liver metastases, and a preponderance of lung and brain metastases. Excluding breast and gastric cancer, the incidence of *HER2*-amplified cancers in the USA is >22,000 per annum.

**Conclusions:**

*HER2* amplification occurs in 2% of PDAC, and has distinct features with implications for clinical practice. The molecular heterogeneity of PDAC implies that even an incidence of 2% represents an attractive target for anti-*HER2* therapies, as options for PDAC are limited. Recruiting patients based on *HER2* amplification, rather than organ of origin, could make trials of anti-HER2 therapies feasible in less common cancer types.

## Background

Pancreatic cancer is the fourth leading cause of cancer death in western societies, with a 5-year survival rate of less than 5% [1]. Systemic therapies are only modestly effective; however, there is emerging evidence that small groups of patients may respond well to specific treatments [2,3]. Current therapeutic development is focused on targeting molecular mechanisms, and this has resulted in significant improvements in outcome for several cancer types (for example, crizotinib for *EML4-ALK* fusion-positive non-small cell lung cancer (NSCLC)). This approach is shifting the traditional organ-based classification of cancer towards a new molecular taxonomy, and creating opportunities to apply therapeutics for the treatment of cancers originating in other organs that harbour similar molecular anomalies. Such indications for extension of existing therapeutics is attractive; however, specific molecular phenotypes and diagnostic characteristics are complex and usually inadequately defined [4,5]. The target population for cancers of organs, apart from where the therapeutic strategy was initially developed, often occur at low frequency, further adding to the challenge.

Emerging data from cancer sequencing initiatives such as the International Cancer Genome Consortium (ICGC) [6] and The Cancer Genome Atlas (TCGA) [7] are unveiling a vast heterogeneity of molecular aberrations in cancer. Pancreatic ductal adenocarcinoma (PDAC), the predominant form of pancreatic cancer, is particularly heterogeneous, and apart from a few notable exceptions, which have not been successfully targeted, most genetic aberrations have a frequency of 2% or less [8-10].

Trastuzumab, a monoclonal antibody that targets the *HER2* receptor, is an effective therapy for *HER2*-amplified breast cancer, and was recently extended to the treatment of *HER2*-amplified gastric cancer [11]. In addition, borderline signals have been seen in clinical trials of semi-selected patients with NSCLC [4], and case reports describe exceptional responses to trastuzumab in other cancers with *HER2* amplification, such as cholangiocarcinoma [12]. Defining *HER*2 amplification as a biomarker of trastuzumab responsiveness is integral to targeting appropriate populations for therapy. Although *HER2* overexpression and amplification has been assessed in PDAC (see Additional file 1), standardized diagnostic assays have on the whole not been applied, and the roles of emerging diagnostic approaches such as genomic sequencing are yet to be explored. As a consequence, the diagnostic criteria and prevalence of *HER2* amplification in PDAC remain unclear. Although preclinical studies support potential efficacy of trastuzumab in PDAC [13,14], clinical trials have been hampered by non-standardized assays and a consequent lack of focus on appropriate subgroups [15,16]. With the current poor survival, low therapeutic responsiveness, and vast molecular heterogeneity of PDAC, even if a relatively low proportion were found to be *HER2*-amplified, targeting by *HER2* amplification represents an attractive potential therapy.

In this study, we identified *HER2* amplification in diagnostic specimens of PDAC using single nucleotide polymorphism (SNP) arrays and whole genome sequencing. We defined the characteristics and prevalence (2.1%) of *HER2* amplification in a large cohort of patients with resected PDAC using standardized reference laboratory assays. We found that *HER2*-amplified PDAC has an atypical pattern of metastatic spread with a predilection for lung metastasis and local recurrence, rather than liver metastases. Assessment of *HER2* amplification across 16 cancer types suggested a prevalence of at least 22,000 cases per annum in the USA (excluding breast and gastric cancer), suggesting that a molecular recruitment strategy may make it feasible to test anti-HER2 therapies in less common cancer types.

## Methods

### Ethics approval

Ethics approval for acquisition of data and biological material was obtained from the human research ethics committee at each participating institution, conducted in accordance with the National Statement on Ethical Conduct in Human Research 2007 and the Declaration of Helsinki. Consent was obtained from prospectively recruited patients for genomic sequencing through the Australian Pancreatic Cancer Genome Initiative (APGI) as part of the ICGC. Consent was waived by Human Research Ethics Committees for retrospectively acquired data and material under an approved protocol (see Additional file 2).

### Genomic sequencing, copy number, and mRNA expression analysis

Patients were prospectively recruited to the APGI [17] for genomic sequencing as part of the ICGC, and details of sample acquisition and processing have been described previously [9]. Briefly, samples focused on primary operable non-pretreated PDAC. Tissue was prepared by either full face frozen sectioning or the ends being excised and processed in formalin, then representative sections were reviewed by at least one pathologist to verify presence of carcinoma in the sample to be sequenced, and to estimate the percentage of malignant epithelial nuclei in the sample relative to stromal nuclei. Nucleic acids were extracted from fresh frozen tumour and normal tissue pairs, and whole genome and exome sequencing was performed using a combination of long mate pair and paired-end approaches. DNA copy number was assessed using specific microarrays (HumanOmni1-Quad BeadChip, Illumina Inc., San Diego, CA, USA). Primary tumour mRNA expression was assayed using human microarrays (HT-12 V4; Illumina) (GEO accession GSE36924). Gene expression profiles were classified into intrinsic breast cancer subtypes using the PAM50 classifier [18].

### Defining prevalence and diagnostic criteria for *HER2* amplification

Formalin-fixed, paraffin wax-embedded diagnostic material from a cohort of 469 patients who had undergone operative resection for PDAC was accrued from 12 institutions associated with the APGI between 1990 and 2012. Subsets of this cohort have previously been published [19,20], and detailed characteristics are presented (see Additional file 3: Table S2).

HER2 immunohistochemistry (IHC) and *in situ* hybridization (ISH) was performed in a national reference HER2 diagnostic testing laboratory. Scoring for *HER2* protein expression and amplification was based on criteria recommended for breast and gastric carcinoma (Table 1) [21].

**Table 1:** **IHC and FISH scoring for the detection of *HER2 *** **amplification**^**a**^

**Her2 IHC score**	**IHC scores**	***HER2 *** **FISH**	
	**(n = 469)**	**Positive, n = 10**	**Negative, n = 459**
3+	7 (1.5%)	7	0
2+	27 (5.8%)	3	24
1+	59 (12.6%)	0	59
0	376 (80.2%)	0	376

Abbreviations: *FISH* fluorescence *in situ* hybridization; *IHC* immunohistochemistry; *PDAC* pancreatic ductal adenocarcinoma.

^a^The criteria used for scoring HER2 by immunohistochemistry and *in situ* hybridization in PDAC were as follows. Her2 IHC score criteria (modified from Hofmann *et al*[21]): 0, no staining of any pattern or intensity; 1+. weak discernable membrane staining; 2+, mild to moderate complete or basolateral membrane staining; 3+, strong, complete, or basolateral membrane staining. *HER2* ISH criteria: non-amplified, Her2 count <4 and Her2:cep17 ratio <2; amplified, Her2 count ≥4 and Her2:cep17 ratio ≥2.

## Results

**Figure 1:**
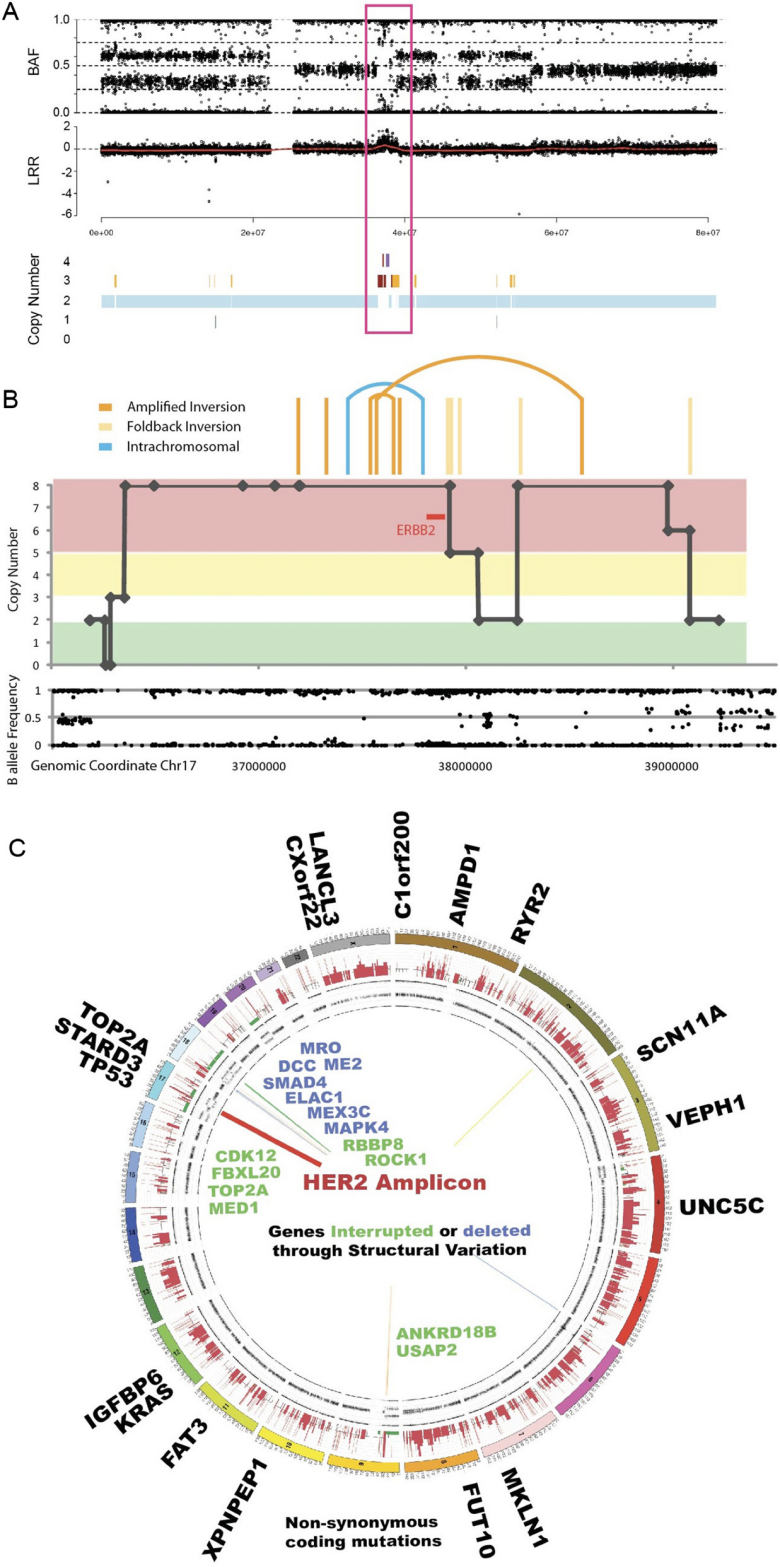
***HER2*-amplified pancreatic ductal adenocarcinoma. (A)** Single nucleotide polymorphism (SNP) array showing amplification on chromosome 17 containing the *HER2* locus (boxed). **(B)** Inverted amplification on chromosome 17, spanning *HER2* and truncating *MED1* and *TOP2A* at either end (chr17:37,565,271-38,554,848). **(C)** Circos plot showing mutations and structural rearrangements of patient 9 (Table 2). Genes with non-silent substitutions are shown on the outer wheel. The SNP array data are shown on the subsequent plots (copy number predictions and B-allele frequency of probes). Structural rearrangements are indicated with lines inside the circle: deletions (green), inversions (yellow), intra-chromosomal rearrangements (blue), foldback inversions (light orange), and amplified inversions (dark orange). Genes with structural variation are shown in blue if deleted and green if interrupted.

In the initial set of 50 patients recruited to the APGI, SNP arrays detected a case of *HER2* amplification (Figure 1A), with associated high *HER2* mRNA expression (see Additional file 4). *HER2* amplification was verified using standardized laboratory assays currently used for breast and gastric cancer at an *HER2* diagnostic reference laboratory. This case was analyzed further using whole genome sequencing, copy number analysis, and mRNA expression profiling.

### Genomic characteristics of *HER2*-amplified pancreatic ductal adenocarcinoma

Whole genome and exome sequencing identified several classes of mutation including single nucleotide variants, small insertions and deletions (indels), and chromosomal rearrangements (see Additional file 5). Chromosomal rearrangements included a region of loss on chromosome 18 containing the *MAPK4*, *DCC*, *SMAD4*, and *ELAC1* genes, and three complex amplification regions: an inverted amplification of a region of chromosome 17, spanning *HER2* and truncating *MED1* and *TOP2A* at either end (chr17:37,565,271-38,554,848) (Figure 1B), and two overlapping fold back inversion events on chromosome 9: a 1.5 Mb event within a 1.9 Mb event, disrupting *UBAP2*. Exome sequencing identified 23 somatic mutations affecting 21 genes, including *KRAS*^p.G12V^ and *TP53*^p.Q317^, and 2 mutations in both *TOP2A* and *RYR2* (Figure 1C).

We performed gene set enrichment analysis using all genes affected by mutation, amplification, copy number alterations (GISTIC2.0 peaks *P* < 0.25), and disruptions caused by structural rearrangements (see Additional file 6). There were three strong biological themes: 1) genes from the *HER2*-amplicon (13 genes; *P*<1 × 10^-7^); 2) a broad cancer theme driven by *KRAS*, *TP53*, and *EPHA5* (13 gene sets; *P*<0.001); and 3) an immune signature, driven by type I interferons, *IFNA2*, *INFA6*, *INFA13* (7 gene sets; *P* < 0.001), which are involved in recognition of viral infection and neoplasia (see Additional file 7).

Intrinsic subtype analysis using expression microarray data from the APGI cohort (n = 90) [9] classified each PDAC sample using the PAM50 classifier, which captures five breast cancer subtypes: *HER2*-amplified, luminal A, luminal B, basal, and normal-like [18]. The *HER2*-amplified patient with PDAC identified above clustered with the *HER2*-amplified intrinsic subtype, with a confidence of 93%. This was driven by high expression of *HER2*, *GRB7*, and *FOXA1*, and low expression of *KRT17* and *MIA* (see Additional file 8).

### *HER2* amplification in pancreatic ductal adenocarcinoma

**Figure 2:**
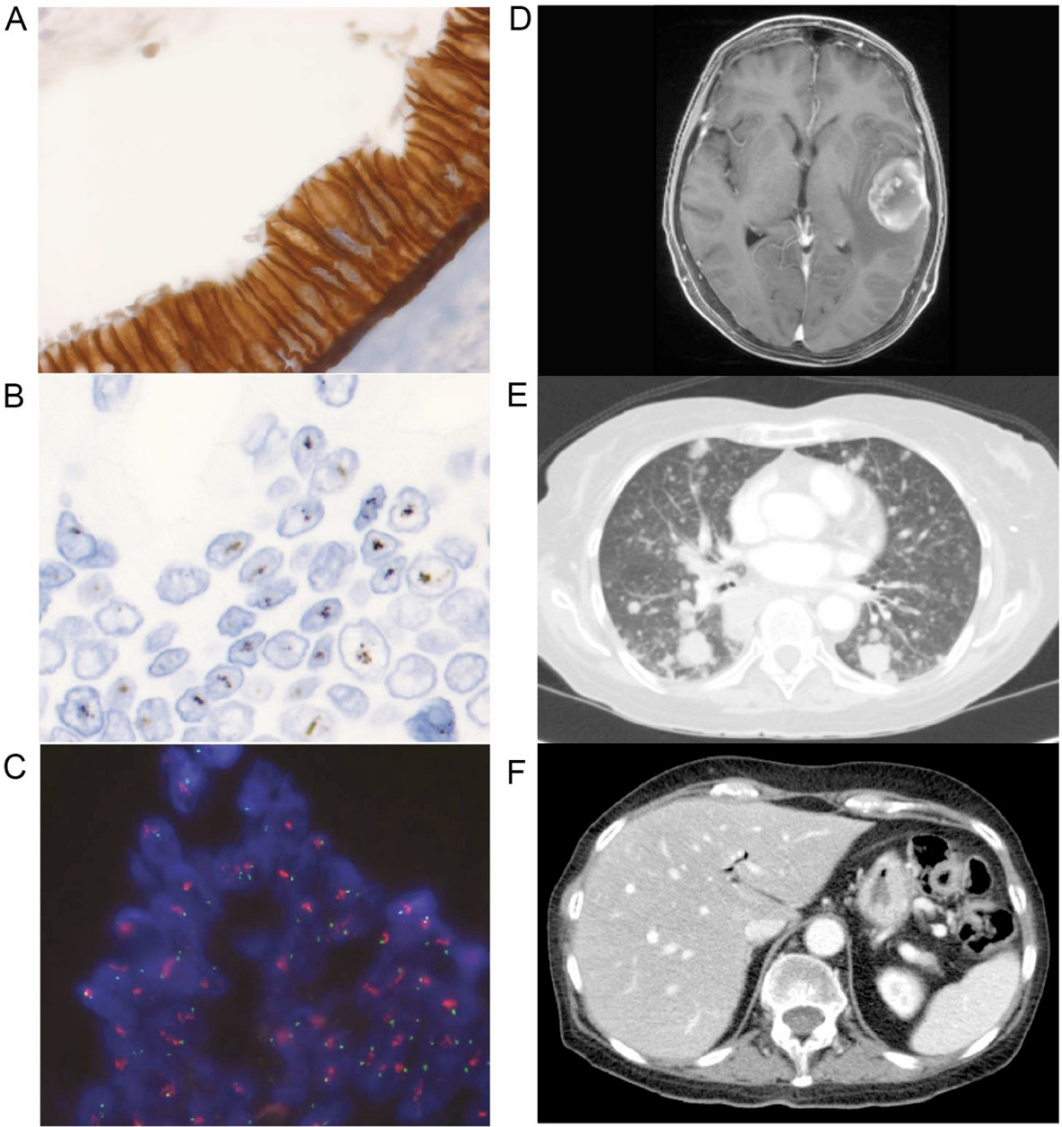
**Clinicopathological features of *HER2*-amplified pancreatic ductal adenocarcinoma (PDAC). (A)** Her2 immunohistochemistry showing 3+ staining, and corresponding *HER2* amplification on **(B)** silver and **(C)** fluorescence *in situ* hybridization. Imaging results of patient 9 (Table 2) showing **(D)** cerebral and **(E)** lung metastases, and **(F)** absence of liver metastases.

In order to define the clinical and histopathological characteristics, diagnostic criteria, and prevalence of *HER2* amplification in PDAC, we assessed a cohort of 469 patients with resected PDAC using IHC and *in situ* hybridization (ISH). IHC for Her2 protein expression showed 3+ staining in 7 cases (1.5%) (Figure 2A), 2+ staining in 27 (5.8%), 1+ in 59 (12.6%) and no staining in 376 (80.2%) (Table 1). All 7 cases that exhibited Her2 3+ staining were found to be *HER2*-amplified on silver ISH and fluorescence ISH (FISH) with 100% concordance between the two methods (Figure 2B,C). Only 3 of 27 cases that were Her2 2+ on IHC were found to be *HER2*-amplified (11%). No cases of 1+ or negative Her2 IHC were *HER2*-amplified, and the overall prevalence of *HER2* amplification was 2.1% (10 of 469 cases). All the amplified cases showed a tendency for the *HER2* signal to form clusters. One case was found to have intra-tumoral heterogeneity by IHC and ISH, with distinct *HER2*-positive and *HER2*-negative areas. As *HER2* amplification was only present when Her2 IHC produced 2+ or 3+ staining, IHC screening followed by ISH in cases of 2+ and 3+ IHC staining may be an appropriate and cost-effective approach for the detection of *HER2*-amplified PDAC (see Additional file 9).

### Clinical features of *HER2*-amplified pancreatic ductal adenocarcinoma

Of the ten patients with *HER2* amplified tumours, nine died of PDAC, and one died of respiratory failure as a result of chronic obstructive pulmonary disease with no evidence of cancer recurrence. The pattern of metastatic relapse of the *HER2*-amplified cases was characterized by a lack of liver metastases. None of the eight cases with a documented site of recurrence had liver metastases (one patient had recurrence, and for another patient the site of recurrence was not reported). Of these eight patients, four had lung metastases (one of whom also had brain metastases (Figure 2D-F), and two also had local recurrence, although cerebral imaging was only performed in one patient), while the remaining four patients had peritoneal and local recurrences only, although imaging of the thorax was not performed in two of these patients (Table 2). The higher prevalence of lung metastasis (in the absence of liver metastases) in *HER2*-amplified tumours was significantly different to that of non-*HER2*-amplified tumours (*P* = 0.0022; Table 3), as was the rate of recurrence at any site without liver metastases (*P* = 0.0028; Table 3). *HER2* amplification was not associated with any other clinicopathological characteristics such as age (*P* = 0.5467), sex (*P* = 0.7520), tumour stage (*P* = 0.4495), grade (*P* = 0.1259), or size (*P* = 0.4695), or disease-specific survival (*P* = 0.2502; Table 3). Histopathologically, there were no specific features to distinguish *HER2*-amplified tumours from non-*HER2*-amplified tumours. All *HER2*-amplified cases were moderately differentiated, and most had a diffuse or at least focally macroglandular architecture.

**Table 2:** **Clinicopathological characteristics of *HER2***-**amplified PDAC**

**Patient**	**Her2 IHC score**	***HER2 *** **ratio**	***HER2 *** **count**	**Overall survival (months)**	**Cause of death**	**Pattern of recurrence**					**Comments**
						**Lung**	**Brain**	**Liver**	**Local**	**Peritoneal**	
1^a^	3+	2.45	6	13.6	PDAC	**-**	ND	**-**	**+**	**+**	Laparotomy showed peritoneal and local recurrence
2	3+	7.5	13	43.5	COPD	**-**	ND	**-**	**-**	**-**	CT at 43 months showed no recurrence
3	3+	4.12	10	5.0	PDAC	**-**	ND	**-**	**+**	**-**	Local recurrence
4^b^	2+	2.5	5.4	36.5	PDAC	**+**	ND	**-**	**+**	**+**	Ascites/pleural effusions, no liver metastases on CT
5	3+	4.38	8.1	18.6	PDAC	ND	ND	ND	ND	ND	Site not documented
6	2	3.56	7	10.1	PDAC	ND	ND	**-**	**+**	ND	R2 resection
7	3+	2.7	4.5	41.6	PDAC	ND	ND	**-**	**+**	**+**	No liver metastases on CT; peritoneal recurrence
8	2+	5.32	9	10.9	PDAC	**+**	ND	**-**	**+**	**-**	No liver metastases on CT
9^c,d^	3+	2.95	6.2	35.0	PDAC	**+**	**+**	**-**	**-**	**-**	No liver metastases on CT
10^e^	3+	2.86*	6.3	27.0	PDAC	**+**	ND	**-**	**-**	**-**	No liver metastases on CT

Abbreviations: + present; - absent; *COPD* chronic obstructive pulmonary disease; *CT* computed tomography; *FISH* fluorescence *in situ* hybridization; *IHC* immunohistochemistry; *ND* not determined; *PDAC* pancreatic ductal adenocarcinoma.

^a^Received adjuvant 5-fluouracil.

^b^Received adjuvant gemcitabine.

^c^Only case 9 received palliative chemotherapy (gemcitabine + erlotinib) and also received adjuvant gemcitabine.

^d^Case that was whole genome sequenced.

^e^Case 10 showed heterogeneous staining.

**Table 3:** **Clinicopathological characteristics of *HER2 *** **amplified and *HER2 *** **non-amplified cases**

**Clinicopathological characteristics**	**Total cohort, n = 469**	**HER2 amplification**		***P*-value**
		**Amplified, n = 10**	**Non-amplified, n = 459**	
Sex, n (%)				
Male	241 (51)	6 (60)	220 (51)	0.7520^b^
Female	228 (49)	4 (30)	210 (49)	
Age, years				
Mean	66	64	66	0.5467^c^
Median	68	69.5	67.5	
Range	28 to 88	47 to 73	28 to 87	
AJCC stage, n (%)				
1a	17 (3.6)	1 (10)	16 (3.5)	0.4495^d^
1b	25 (5.3)	1 (10)	24 (5.2)	
2a	132 (28.1)	3 (30)	129 (28)	
2b	279 (59.5)	4 (40)	275 (60)	
4	16 (3.4)	1 (10)	15 (3)	
T stage, n				
T1	32	1	31	
T2	64	2	62	
T3	372	7	365	
T4	1	0	1	
N stage, n				
N0	180	6	174	
N1	289	4	185	
AJCC grade, n				
1 (well differentiated)	36	0	36	0.1259^d^
2 (moderately differentiated)	305	10	295	
3 (poorly differentiated)	125	0	125	
4 (undifferentiated)	3	0	3	
Tumour size, mm				
<=20	105	3	102	0.4695^b^
>20	364	7	357	
Vascular invasion, n				
Present	222	2	220	0.1106^b^
Absent	247	8	239	
Perineural invasion, n				
Present	339	8	331	0.7336^b^
Absent	130	2	128	
Tumour location, n				
Head	381	9	372	0.6961^b^
Others	88	1	87	
Margins				
Clear	301	7	294	1.0000^b^
Involved	168	3	165	
Outcome				
Follow-up, months	0.03 to 240	5.0 to 43.6	0.03 to 240	1.0000^b^
Median follow-up, months	16	23	16	
Death: PDAC	369	8	361	
Death: other	32	2	30	
Death: unknown	16	0	16	
Alive	49	0	49	
Lost to follow-up	3	0	3	
Cancer-specific survival, mean ± SD				
Length, months	20 ± 19.43	28 ± 19.65	20 ± 19.55	0. 2502^c^
Recurrence				
Present	261	8	253	
Absent	78	1	77	
Unknown	130	1	129	
Pattern of recurrence, n (% of total n)				
Lung without liver metastasis	22 (8.4% of 261)	4 (50% of 8)	18 (7.1% of 253)	0.0022^b^
Any recurrence without liver metastasis	127 (49% of 261)	8 (100% of 8)	119 (47% of 253)	0.0028^b^
Adjuvant therapy, n				
Yes	175	3	172	
No	289	7	282	
Unknown	5	0	5	

AJCC, American Joint Committee on Cancer; PDAC, pancreatic ductal adenocarcinoma.

^a^AJCC Cancer Staging Manual, seventh edition.

^b^Fisher’s exact test.

^c^Unpaired *t*-test.

^d^χ^2^ test.

Since the completion of this study, we have begun prospectively screening patients with PDAC for *HER2* amplification using this diagnostic approach, and identified a further two individuals (of eleven) with *HER2* amplification. The first was a primary resected cancer with no evidence of recurrent disease to date at 6 months. Subsequently, whole genome sequence analysis on this patient’s tumour confirmed mutation in KRAS ^*p.G12V*^. The second patient had an atypical pattern of metastatic disease at diagnosis, with a small primary pancreatic tumour on imaging, histologically confirmed peritoneal disease in the pelvis, and nodules in the lungs. Because of the unusual pattern of disease, PDAC was verified using a targeted exome panel of tissue from this patient’s peritoneal disease, which showed mutations in *KRAS*^*p.G12V*^ and *SMAD4*, further supporting the diagnosis of PDAC.

### *HER2* amplification in other cancer types

The low prevalence of HER2 amplification in PDAC and many other cancer types makes clinical trials challenging. Analysis of genomic sequence data for 16 other cancer types (by TCGA, ICGC and other large-scale genomic efforts) identified an incidence of 0.5% to 13%, which equates to over 54,000 new cases of *HER2*-amplified cancers per year in the USA alone (see Additional file 10). Excluding breast and gastric cancer, we estimate an incidence in excess of 22,000 *HER2* amplified cancers per annum. Recruiting patients based on *HER2* amplification rather than organ of tumour origin would make trials of anti-HER2 therapies feasible in less common cancer types. In order to identify these 22,000 patients, 740,000 patients would need to be screened using IHC, and of these, all 2+ and 3+ IHC cases (~8%) would require validation using ISH. This approach may circumvent the challenges of previous clinical trials based on organ of origin recruitment, in which there have been mixed results but some efficacy in individuals (see Additional file 11). An orthogonal approach by which a primary molecular classification is used and patients are sub-classified based on organ of origin may be more tractable.

## Discussion

In this study, we found that *HER2*-amplified PDAC has a prevalence of 2%, is detectable using contemporary genomic approaches, is associated with a clinical phenotype characterized by metastatic spread predominantly to the lungs and peritoneum with local recurrence, and can metastasize to the brain but tends to avoid the liver. It bears molecular similarities to *HER2*-amplified breast cancer, and is yet to be adequately assessed for potential responsiveness to anti-HER2 therapy. Multiple studies in large cohorts have shown that *HER2*-amplified breast cancers more commonly metastasize to the lung and the brain [22]. In PDAC, clinical trial data indicate that the first site of distant metastases occurred most commonly in the liver (50%), and occurred in the lung in 9% of the cases [23]. Autopsy studies have reported that 80% of distant metastases are to the liver, which occurred either alone or in combination with peritoneal and or lung metastases, while metastases sparing the liver made up the rest, and occurred in the peritoneum, lung, adrenal glands, and lymph nodes [24]. Cerebral metastases were not found in these studies. In our cohort of 469 patients, the incidence of lung metastases without liver metastases was 8%, comparable to previous studies. We detected only one case with brain metastases (0.2%), although only this single patient was investigated specifically for brain metastases. If we include all 10 *HER2*-amplified cases with documented metastatic disease, none had evidence of liver metastases (*P* = 0.0028), and the rate of lung metastases was 50% (*P* = 0.0022). These data suggest that *HER2*-amplified PDAC may have a distinct clinical phenotype, and that liver metastases are not determined by physical factors such as portal blood flow, but by the pathophysiology of disease.

These findings have significant clinical implications. First, the detection of small lung nodules should not delay the diagnosis of metastatic disease originating in the pancreas or at relapse if the liver and other sites are clear, particularly in known *HER2*-amplified cases. Second, if an individual is known to have an *HER2*-amplified PDAC, then monitoring for disease progression in non-traditional sites such as the lung and the brain, with vigilance for neurological symptoms may be prudent. Finally, there is potential for anti-HER2 therapies in this subset of patients.

*In situ* hybridization studies in our reference laboratory identified *HER2* amplification only in PDACs with high protein expression by IHC (score 2+/3+). Therefore, a reasonable and cost-effective approach to universal *HER2* screening is to initially test all cases with IHC and then perform secondary ISH testing only on cases with 2+/3+ staining, as was initially performed for breast cancer. Using this approach, 8% of PDAC (2+ and 3+ cancers) will require *HER2* ISH assessment, and of these one-quarter will be amplified.

In the current study, in-depth genomic analysis, apart from *HER2* amplification, did not reveal any features that are atypical of PDAC, with mutations of *KRAS* and *TP53* and loss of *SMAD4* found, although the inherent heterogeneity of PDAC makes it difficult to draw conclusions about the other mutations detected. mRNA expression profiles clustered with *HER2*-amplified breast cancer, suggesting that *HER2* may be an important driver of carcinogenesis in this subgroup of PDAC.

It is interesting to note that all three *HER2*-amplified cases with available genomic data harboured the *KRASp.G12V* mutation. This mutation is less common than the p.G12D mutation, and accounts for 32% of *KRAS* mutations in PDAC versus 40% for p.G12D [9]. Given the small numbers of *HER2*-amplified cases, further studies of larger cohorts will be required before it can be determined if this association is sufficiently robust to be used diagnostically or targeted therapeutically.

Two clinical trials have assessed targeted trastuzumab therapy in PDAC [15,16]. Both are single arm phase II trials used in combination with gemcitabine [15] and capecitabine [16]. Although the latter performed *HER2* FISH for 2+ expressing cases, the former did not, and neither verified the 3+ IHC cases by FISH. In addition, these were not standardized assays performed in reference laboratories, and resulted in a HER2 positive rate of over 10%. This likely overestimation underpowered the trials by over 80%, making a negative result uninterpretable.

## Conclusion

*HER2* amplification occurs in 2.1% of PDAC cases, and is associated with an atypical pattern of metastatic disease. A number of cancers of different organs including NSCLC, ovarian cancer, cholangiocarcinoma, and PDAC have well-defined low-prevalence *HER2* amplification. Testing anti-*HER2* therapies may not be feasible in organ groups because of this low prevalence and the likely heterogeneous response rates. However, these studies could be approached using novel adaptive clinical trials testing personalized therapeutic strategies (for example,: BATTLE [25], I-SPY [26], FOCUS 4 [27] and IMPaCT [3]), or using a molecular taxonomy or ‘biotype’ that recruits *HER2*-amplified cancers irrespective of the organ in which the tumour arises (often referred to as ‘basket’ trials) [28], with specific attention to diagnostic criteria for patient recruitment, particularly as more effective anti-*HER2* therapies emerge.

## Abbreviations

APGI: Australian Pancreatic Genome Initiative; FISH: Fluorescence *in situ* hybridization; ICGC: International Cancer Genome Consortium; IHC: Immunohistochemistry; ISH: *In situ* hybridization; NSCLC: Non-small cell lung cancer; PDAC: Pancreatic ductal adenocarcinoma; SNP: Single nucleotide polymorphism; TCGA: The Cancer Genome Atlas.

## Competing interests

JW has received honoraria received from Roche; RS has received clinical research funding received from Therapeutic Innovation Australia and Cancer Council New South Wales; DG has an uncompensated consultant/advisory role with Celgene, Bayer, and Pfizer, and has received clinical research funding from Celgene, Pfizer, and Amgen; AM is on the Roche HER2 advisory board in Australia, and had received honoraria from Roche. The remaining authors declare that they have no competing interests.

## Authors' contributions

AC, NW, MJC, AJG, DKC, JW, MP, ALJ, AMN, CT, VC, LC, JH, JSS, ALM, SMG, and AVB were responsible for the concept and design of the study. All authors participated in collection and assembly of data, and AC, NW, MJC, AJG, DKC, JW, KK, MP, DG, ALM, SMG and AVB were involved in data analysis and interpretation. AJ, APGI, LC, HW, JS, AG, DKC and AVB were involved in provision of study materials or patients: All authors were involved in manuscript writing and final approval of the manuscript.

## Supplementary Material

Additional file 1: Table S1 Literature reports of *Her2* positive rate in pancreatic ductal adenocarcinoma by immunohistochemistry and *in situ* hybridization techniques. Click here for file

Additional file 2: Supplementary methods Click here for file

Additional file 3: Table S2 Clinico-pathological characteristics of the cohort. Click here for file

Additional file 4: Figure S1 HER2 mRNA expression relative to cohort. Click here for file

Additional file 5: Table S3 Single nucleotide variations (SNVs) and small insertions or deletions (indels) within a patient with HER2 amplification, identified by whole exome sequencing, in MAF format. **Table S4.** Single nucleotide variations (SNVs) and small insertions or deletions (indels) annotated and compared with data from dbSNP, COSMIC, TCGA Breast, Biankin *et al*. [9] and Jones *et al*. [10]. Non-synonymous mutations were assessed for functional effects by two algorithms, Polyphen2 and SIFT; scores close to 1.0 and 0.0, respectively, are predicted to be probably damaging. These data have already been imported into COSMIC, thus many of the COSMIC identification numbers are unique only to this patient. **Table S5:** Genes affected by structural variation, as determined by whole genome sequencing of tissue from a patient with HER2 amplification. **Table S6:** Structural and copy number aberrations in a patient identified with HER2 amplification. Listed here are the genes affected by structural variation (SV) as determined by whole genome sequencing, or copy number altered, as determined by single nucleotide polymorphism (SNP) array analysis. Genes of interest were defined as those genes affected by SV that were expected to have a functional effect (that is, genes that were truncated, amplified (but not duplicated) or deleted); genes whose copy number was found by SNP array analysis to be greatly altered, that is, reduced to 0, or the algorithm’s maximum of four copies (see Methods); and genes that were affected by copy number variation and that overlapped the statistically significant copy number altered regions, as determined by GISTIC 2.0 analysis (see Biankin *et al*., [9]), reported here by their q-value. Click here for file

Additional file 6: Table S7 Genomic alterations within a patient identified with HER2 amplification. Genes of interest were defined as those affected by non-synonymous single nucleotide variation (SNV); small insertions or deletions (see Additional file 5: Table S3); genes affected by structural variation (SV) as determined by whole genome sequencing, or copy number variation (CNV) as determined by SNP array analysis (see Additional file 5: Table S6). Click here for file

Additional file 7: Table S8 MSigDB analysis of all genes affected by mutation, tructural variation (SV) and copy number variation (CNV) (from Additional file 6: Table S7). Click here for file

Additional file 8: Figure S2 Gene expression profiles from 90 pancreatic cancer primary tumors, classified into five intrinsic breast subtypes by the PAM50 classifier. The HER2-amplified patient is highlighted by a white box; genes overexpressed or underexpressed in this patient are highlighted in red and green, respectively. Click here for file

Additional file 9: Figure S3 Algorithm for diagnostic testing for *HER2* amplification. Click here for file

Additional file 10: Table S9 Incidence of and deaths from *HER2*-amplified cancers in the USA. Extrapolating from the frequency of *HER2* amplifications identified from current projects from The Cancer Genome Atlas (TCGA) and our own International Cancer Genome Consortium (ICGC) pancreatic ductal adenocarcinoma (PDAC) project, with the estimated number of new cancer cases in the USA in 2012. From the 1.37 million expected cases, we would expect 54,000 (3.94%) new cases of HER2-amplified cancer. Excluding breast and stomach, for which *HER2* diagnostic assays are common practice, we expect 740,000 new cases, 22,000 of which will be *HER2*-amplified. Extrapolating from the number of immunohistochemistry (IHC) and *in situ* hybridization (ISH) assays to identify *HER*2-amplified PDAC (see Additional file 9: Figure S3), to identify these additional cases, we would expect to perform 742,000 IHC and 117,000 FISH assays, of which 22,000 will be confirmed (that is, will have IHC staining of 2+ or 3+ and be ISH-positive). **Figure S4.** Prevalence of *HER2* amplification (≥4 copies) in cancer: data from The Cancer Genome Atlas (TCGA), via the cBio portal, and the International Cancer Genome Consortium (ICGC) [9]. Click here for file

Additional file 11: Table S10 Clinical trials of *HER2*-amplified cancers, excluding breast and stomach (gastric) cancers. Click here for file
