# Supplementary material for: Clinical and molecular characterization of HER2 amplified-pancreatic cancer
Source: Genome Med. 2013 Aug 31;5(8):78. doi: 10.1186/gm482 (PMC3978667; doi:10.1186/gm482)
Supplement: Additional file 2: Supplementary methods [file gm482-S2.docx]

**Supplementary Methods**

Patients were prospectively recruited to the Australian Pancreatic Cancer Genome Initiative (APGI; [www.pancreaticcancer.net.au](http://www.pancreaticcancer.net.au)) for genomic sequencing as part of the International Cancer Genome Consortium (ICGC). We recently reported exome-sequencing of a subset of this cohort[^1^](#_ENREF_1), hereafter we refer to this as the APGI cohort.

**Exome sequencing, copy number and mRNA expression analysis**

DNA was extracted from fresh frozen tumour-normal pairs for exome sequencing, and SNP array copy number analysis. Exome sequencing of DNA captured with the SureSelect Whole Exome protocol (Agilent) was performed using a SOLiD v4 sequencer (Applied Biosystems), somatic single nucleotide variants and indels were identified with sufficient tumor cellularity using qSNP (manuscript submitted) and the Bioscope Small Indel Tool (Life Technologies). DNA was assayed with the HumanOmni1-Quad BeadChip as per manufacturer’s instructions (Illumina, San Diego CA). Somatic copy number alterations were identified from tumor-normal pairs using GenoCN,[^2^](#_ENREF_2) and confirmed with Partek Genomics Suite, version 6.6 (Partek Inc., St Louis, MO, USA). Primary tumor mRNA was assayed using human HT-12 V4 microarrays (Illumina, San Diego CA), with variance stabilization and robust spline normalization. *HER2* has 3 microarray probes: ILMN_1728761, ILMN_1717902 and ILMN_2352131, all of which perfectly & uniquely match their intended target, as assessed using BLAT[^3^](#_ENREF_3) and a probe re-annotation pipeline.[^4^](#_ENREF_4)

**Long mate pair sequencing and analysis of structural rearrangements**

For the patient with *HER2* amplification, a long mate-pair library was made according to Applied Biosystems Mate-Pair Library Preparation 5500 Series SOLiD^TM^ Systems protocol using 5ug of DNA which was sheared using the Covaris®S220 System, then sequenced using the SOLiD platform (Applied Biosystems). Sequence data was mapped to Genome Reference Consortium GRCh37 assembly using bioscope v1.2.1 (Applied Biosystems). The tumor and matched normal sample was sequenced to an average non-redundant physical coverage of 75 and 100 respectively. Structural rearrangements were determined by analyzing clusters of discordant read pairs using an in-house tool qSV (manuscript in preparation). Depending on the read pair types supporting an aberration or the association of copy number data from the SNP arrays[^5^](#_ENREF_5), events were classified as: deletions, duplications, tandem duplications, inversions, fold back inversions, amplified inversions, intra-chromosomal rearrangements or inter-chromosomal translocations. Data was visualized with circos[^6^](#_ENREF_6) and genes that were impacted by structural rearrangements were annotated using ENSEMBL v61.

**Intrinsic subtype classification**

Gene expression profiles from 90 patients from the APGI cohort were classified into intrinsic breast cancer subtypes using the PAM50 classifier[^7^](#_ENREF_7). Because the PAM50 classifier is sensitive to normalization,[^8^](#_ENREF_8) we performed additional median-normalization, and restricted further analysis to the 50 genes within the PAM50 classifier, selecting the most variable probe to represent each gene. The classifier is available at <https://genome.unc.edu/pubsup/breastGEO/clinicalData.shtml>.

**Extended Patient cohort**

An extended cohort of 469 patients with formalin-fixed paraffin-embedded diagnostic material who underwent operative resection for PDAC with curative intent was accrued from 12 medical institutions associated with the APGI between 1990 and 2012. Data was acquired from clinical notes, imaging reports, surgeon’s operative reports and physician correspondence. Data was initially collected retrospectively but became prospective from 2006. The date and cause of death was obtained from Cancer Registries and treating clinicians. Ethical approval for the study was obtained from the Human Research Ethics Committee at each participating institution. All cases underwent central pathology review by at least one specialist pancreatic histopathologist (AC, AG) and non-PDAC subtypes of pancreatic cancer were excluded. Tumor grade and stage were classified using the updated 2010 AJCC/TNM criteria for PDAC.^9^

National sites ethics approval numbers:

- Sydney South West Area Health Service Human Research Ethics Committee, Western Zone, protocol number 2006/54
- Sydney Local Health District Human Research Ethics Committee, protocol number X11-0220
- Northern Sydney Central Coast Health Human Research Ethics Committee, protocol number 0612-251M
- Sydney West Area Health Service Human Research Ethics Committee (Westmead Campus), protocol number HREC2002/3/4.19
- South East Sydney Illawarra Area Health, Northern Hospital Network HREC- protocol number 05/321
- South East Sydney Illawarra Area Health HREC- Southern Section, protocol number 05/54
- St John of God Hospitals Subiaco & Murdoch:  385
- Fremantle Hospital:  09/324
- Royal Adelaide Hospital:  091107a
- Flinders Private Hospital:  167/10
- Austin Hospital:  H2011/04083
- Princess Alexandra Hospital:  09/QPAH/220
- Greenslopes Private Hospital:  09/34

**Immunohistochemistry and in situ hybridisation**

Her2 immunohistochemitry (IHC) and in situ hybridization for *HER2* amplification was performed in a national reference HER2 diagnostic testing laboratory. IHC was performed on formalin-fixed paraffin-embedded tissue microarrays (TMA) containing 3 x 1mm cores using the Ventana Ultra automated staining platform with an FDA approved system for the detection of Her2 expression in breast cancer (anti-Her2 rabbit monoclonal primary antibody, Ventana 4B5 clone). Scoring for Her2 protein expression by IHC was adapted from criteria recommended for assessment of Her2 expression in gastric carcinoma, and was based on the intensity and pattern of membranous staining.^10^ The rationale for this approach was that *HER2* amplified PDAC, like gastric cancer, shows membranous baso-lateral staining with lack of apical staining.

In-situ hybridization using both silver enhanced (single probe Ventana INFORM SISH with Ventana XT overnight hybridization) and fluorescence enhanced (Abbott Pathvysion HER2/cep17 Probe kit with overnight hybridization) techniques were performed on TMAs to asses mean copy number and *HER2* to cep17 ratios by counting at least 20 tumor nuclei. *HER2* amplification was defined as a Her2/cep17 ratio of ≥ 2 AND a Her2 count of ≥4 (Table 1). This approach decreases inter-observer variability when applied to *HER2* testing of gastric/gastro-oesophageal junction cancers.^11^ Most importantly, cases with aneuploidy/polysomsy were excluded. In-situ hybridization was performed in a high volume central referral laboratory for *HER2* testing. All cases with Her2 2+ and 3+ immunostaining detected on tissue microarrays had Her2 IHC and in-situ hybridization repeated on whole sections to assess for heterogeneity of *HER2* amplification.

**References**

1. Biankin AV, Waddell NJ, Kassahn KS et al: **Pancreatic cancer genome reveal aberrations in axon guidance pathway genes.** Nature 2012, 491:399-405.

2. Sun W, Wright FA, Tang Z, et al: **Integrated study of copy number states and genotype calls using high-density SNP arrays.** Nucleic Acids Res 2009, 37:5365-77.

3. Kent WJ: **BLAT--the BLAST-like alignment tool**. Genome Res 2002, 12:656-64.

4. Barbosa-Morais NL, Dunning MJ, Samarajiwa SA et al: **A re-annotation pipeline for Illumina BeadArrays: improving the interpretation of gene expression data.** Nucleic Acids Res 2010, 38:e17.

5. Popova T, Manie E, Stoppa-Lyonnet D, Rigaill G, Barillot E, Stern MH: **Genome Alteration Print (GAP): a tool to visualize and mine complex cancer genomic profiles obtained by SNP arrays.** Genome Biol 2009, 10:R128.

6. Krzywinski M, Schein J, Birol I, et al: **Circos: an information aesthetic for comparative genomics.** Genome Res 2009, 19:1639-45.

7. Parker JS, Mullins M, Cheang MC, et al: **Supervised risk predictor of breast cancer based on intrinsic subtypes.** J Clin Oncol 2009, 27:1160-7.

8. Sorlie T, Borgan E, Myhre S, et al: **The importance of gene-centring microarray data.** Lancet Oncol 2010, 11:719-20; author reply 20-1.

9. Buhimschi IA, Buhimschi CS, Weiner CP, et al: **Proteomic but not enzyme-linked immunosorbent assay technology detects amniotic fluid monomeric calgranulins from their complexed calprotectin form.** Clin Diagn Lab Immunol 2005, 12:837-44.

10. Hofmann M, Stoss O, Shi D, et al: **Assessment of a HER2 scoring system for gastric cancer: results from a validation study.** Histopathology 2008, 52:797-805.

11. Fox SB, Kumarasinghe MP, Armes JE, et al: **Gastric HER2 Testing Study (GaTHER): an evaluation of gastric/gastroesophageal junction cancer testing accuracy in Australia.** Am J Surg Pathol 2012,36:577-82.
